# Personalised depression forecasting using mobile sensor data and ecological momentary assessment

**DOI:** 10.3389/fdgth.2022.964582

**Published:** 2022-11-18

**Authors:** Alexander Kathan, Mathias Harrer, Ludwig Küster, Andreas Triantafyllopoulos, Xiangheng He, Manuel Milling, Maurice Gerczuk, Tianhao Yan, Srividya Tirunellai Rajamani, Elena Heber, Inga Grossmann, David D. Ebert, Björn W. Schuller

**Affiliations:** ^1^ EIHW – Chair of Embedded Intelligence for Health Care and Wellbeing, University of Augsburg, Augsburg, Germany; ^2^ Psychology & Digital Mental Health Care, Technical University of Munich, Munich, Germany; ^3^ Clinical Psychology & Psychotherapy, Friedrich-Alexander-University Erlangen-Nuremberg, Erlangen, Germany; ^4^ GET.ON Institut für Online Gesundheitstrainings GmbH/HelloBetter, Hamburg, Germany; ^5^ GLAM – Group on Language, Audio, & Music, Imperial College London, London, UK

**Keywords:** depression, forecasting, personalised models, machine learning, mHealth, mental illness

## Abstract

**Introduction:**

Digital health interventions are an effective way to treat depression, but it is still largely unclear how patients’ individual symptoms evolve dynamically during such treatments. Data-driven forecasts of depressive symptoms would allow to greatly improve the personalisation of treatments. In current forecasting approaches, models are often trained on an entire population, resulting in a general model that works overall, but does not translate well to each individual in clinically heterogeneous, real-world populations. Model fairness across patient subgroups is also frequently overlooked. Personalised models tailored to the individual patient may therefore be promising.

**Methods:**

We investigate different personalisation strategies using transfer learning, subgroup models, as well as subject-dependent standardisation on a newly-collected, longitudinal dataset of depression patients undergoing treatment with a digital intervention (N=65 patients recruited). Both passive mobile sensor data as well as ecological momentary assessments were available for modelling. We evaluated the models’ ability to predict symptoms of depression (Patient Health Questionnaire-2; PHQ-2) at the end of each day, and to forecast symptoms of the next day.

**Results:**

In our experiments, we achieve a best mean-absolute-error (MAE) of 0.801 (25% improvement) for predicting PHQ-2 values at the end of the day with subject-dependent standardisation compared to a non-personalised baseline (MAE=1.062). For one day ahead-forecasting, we can improve the baseline of 1.539 by 12% to a MAE of 1.349 using a transfer learning approach with shared common layers. In addition, personalisation leads to fairer models at group-level.

**Discussion:**

Our results suggest that personalisation using subject-dependent standardisation and transfer learning can improve predictions and forecasts, respectively, of depressive symptoms in participants of a digital depression intervention. We discuss technical and clinical limitations of this approach, avenues for future investigations, and how personalised machine learning architectures may be implemented to improve existing digital interventions for depression.

## 1. Introduction

Depressive disorders are very common in the general population, with twelve month prevalence estimates ranging from 5.9–7.7% (1–4). They are associated with a vast array of negative consequences for the individual and society, including increased disability (5), loss of quality of life (6, 7), suicidality (8, 9), excess mortality (10, 11), as well as large economic costs (12, 13). By 2030, depression is estimated to become the leading cause of disability-adjusted life years in high-income countries (14, 15). Thus, the development and widespread dissemination of effective treatments for depressive symptoms constitutes a public health priority.

Research documents a substantial treatment gap among individuals who suffer from depression. Even in high-income countries, depression treatment rates are often below 30% (16, 17). It has been found that structural barriers, but also attitudinal factors such as preference to self-manage or personal stigma may play a role in the limited utilisation of existing services (18). Digital interventions have therefore been discussed as an instrument to increase help-seeking (19, 20), since such interventions are easily accessible and can provide greater anonymity. Digital interventions can be provided to everyone with Internet access and are highly scalable. Therefore, they may also allow to address structural barriers in the health care system (21).

Digital interventions have been found to be an effective treatment for a broad range of mental disorders (22), including depression (23). However, it is still largely unknown for whom these interventions work, and why (24). Overall, treatment non-response remains a pervasive issue in major depressive disorder (MDD) patients, with approximately 37% achieving remission after the first course of evidence-based treatment (i.e., pharmacotherapy, psychological interventions, or combination therapy), and 67% after trying out several courses of treatment (25). “Sudden losses” and relapse remain a frequent phenomenon within or following depression treatments (26, 27).

These findings underline the potential of a more personalised treatment approach, particularly with respect to methods that allow for an early detection of symptom changes. Current digital interventions typically follow a “one size fits all” approach that is very limited in its capability to react adaptively to patients’ individual trajectory. Therefore, data-driven methods are increasingly discussed as a method to open up the “black box” of psychological treatment, and thus building the basis for tailored interventions (21, 28). Interventions based on digital applications may be particularly suited for data-driven tailoring, since they allow to capture an unprecedented amount of potentially meaningful symptom and process information. This could allow, for example, to provide targeted behavioral prompts, ecological momentary interventions (29), and additional human guidance when patients experience symptom spikes during treatment; or to exploit predicted improvements by encouraging patients to reflect on potential behavior changes they have since implemented.

There are several data sources by which data-driven models of symptom trajectories have been developed in previous research. In an emerging research field known as “Personal Sensing” (30) or “Digital Phenotyping” (31, 32), scientists are using mobile sensor data as created by commercially available smartphones or wearables to measure high-level indicators of individuals’ mental health (e.g., sleep patterns, stress, or depressed mood). This type of research has established that passive sensor data can be used to predict various symptoms of mental disorders or mental health problems (23, 33–38), as well as their future development [e.g., (39)].

A related research field involves the use of “Ecological Momentary Assessment” [EMA; (40)], in which patients’ behaviors and experiences are repeatedly sampled *in situ*, often using (e.g., digitally administered) self-report questionnaires. EMA is frequently used to gain a personalised understanding of the temporal dynamics of mental disorders, and their influences (41). However, EMA data has also been successfully used to predict the development of symptoms within treatments [e.g., (42)].

Currently, most data-driven approaches employ “general” models to predict current or future mental health symptoms. This means that one model capturing overall patterns across patients is derived from the training set, which allows to generate individualised predictions conditional on provided data. However, it has been recognised that the inherent clinical heterogeneity associated with mental disorders may necessitate a greater focus on inter-individual differences to ensure generalisability to unseen cases (39, 43, 44). With respect to passive sensor data, for example, a large-scale study by (45) demonstrated that depression prediction accuracies using a heterogeneous student sample (n=57) did not generalise once models were applied to a more representative, heterogeneous sample (n=5,262).

Therefore, personalised models are progressively explored as a promising approach to predict mental health in heterogeneous real-world datasets (39, 46, 47). In a personalised learning approach, an idiographic model is developed for each individual or a smaller patient subset, often while retaining a “backbone” infrastructure that captures common features across subjects. Most of the personalisation approaches can be assigned to one of the following three areas: (1) user-specific, (2) similarity-based, as well as (3) enrolment-based approaches.

The first group represents a user-dependent method that depends on data of the user for whom the model will be personalised (46, 48). Busso et al. (49), for example, propose a user-dependent personalisation approach for speech applications using speaker-dependent feature normalisation. Other methods of user-dependent personalisation are approaches that have a common backbone model trained on the whole population, which is extended with personalised layers per subject (46, 48). Similarity-based and enrolment-based methods are user-independent and can be applied even if no longitudinal data of participants is available (50–52). Dividing patients into subgroups makes it possible to train a separate model for each cluster using similarity-based personalisation. By subsequently assigning new patients to the group they are most similar to, the model can learn subgroup-specific characteristics, often leading to an improved performance compared to general trained models. Li and Sano (51), for example, found that user-dependent models perform better in comparison to user-independent approaches. Finally, enrolment-based strategies attempt to adapt to new users using only a limited number of enrolment samples for which the ground-truth label is available (52).

Overall, personalisation may also allow to address frequently overlooked issues concerning the *fairness* of prediction models across patients. Predictive models, especially general ones, often work better in one subgroup of the population than in others. Such biases may further reinforce existing disparities in health care, for example with respect to gender or minority status (53). Model personalisation may also be an auspicious approach to meet this challenge, by allowing to pay greater attention to the variation of predictive accuracies between and within individuals.

In addition to group-level fairness, individual fairness arises in several tasks where the target manifests itself differently in distinct (human) subjects. This ranges from the expression of fatigue in runners (54), to stress and emotion in voice (55, 56), and, crucially, mood prediction from wearable sensors (46). We note that the standard individual fairness formulation proposed by Dwork et al. (57), which purports that similar individuals should receive similar outcomes, is not applicable in our work. This is because Dwork et al. (57) applied their framework to tasks where each individual is assigned once to a particular class (e.g., in recidivism cases); however, we apply our models several times to each individual to predict their PHQ-2 scores over time.

In this study, we therefore examine the utility of different personalisation strategies in providing daily predictions and forecasts of depressive symptoms. We focus on a real-world sample of patients suffering from clinically relevant levels of depression who provided mobile sensor and EMA data while participating in a digital depression intervention. In this context, we also assess the group- and individual-level fairness of the developed approaches. Even though individual fairness emerges in several prior works, there is no widely-accepted metric to quantify it. Therefore, we attempt to bridge this gap by proposing a set of indices motivated by related fields.

## 2. Methods

### 2.1. Data

In this section, we introduce the novel “Mobiler Alltagstherapieassistent mit interaktionsfokussierter künstlicher Intelligenz bei Depression” (MAIKI; german translation for “Mobile Therapy Assistant for Daily Life with Interaction-focused Artificial Intelligence for Depression”) dataset. The MAIKI dataset was collected as part of a prospectively registered feasibility trial within the MAIKI project (German Clinical Trials Register; DRKS00024718). The study procedures have been approved by the ethics committee of the Friedrich-Alexander-University Erlangen-Nuremberg (385_20B).

**Figure 1:**
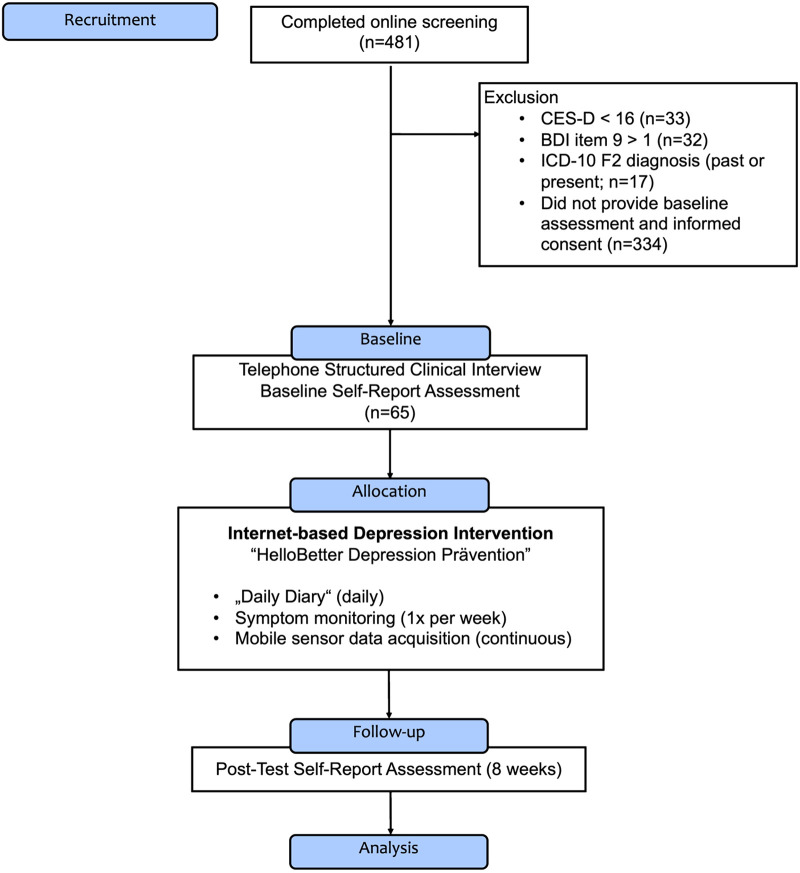
Flow diagram of the “MAIKI” study.

Figure 1 displays a Consolidated Standards of Reporting Trials [CONSORT; (58)]-type flow diagram of the MAIKI trial. Between May 2021 and September 2021, a total of N=65 patients were recruited for the study. Individuals were eligible for the study if they showed elevated symptoms of depression, defined by a score of ≥16 on the 20-item German version of the Center for Epidemiological Studies’ Depression Scale [CES-D; (59)]. Furthermore, participants were required to have access to an Android smartphone. Patients were assessed at baseline and post-test (8 weeks after treatment assignment), resulting in a study period of approximately 8 weeks for each individual. At baseline, the Structured Clinical Interview for DSM-5^®^ Disorders [SCID; 5th Edition; (60)] was conducted by trained psychologists via telephone to determine if patients fulfilled the diagnostic criteria of a manifest depressive disorder. While the SCID-5 is intended to be delivered face-to-face, interviews conducted via telephone have been found to show only slightly inferior reliability, supporting their use for research purposes (61). Participants were then assigned to “HelloBetter Depression Prävention,” a digital depression application. The intervention is based on “GET.ON Mood Enhancer,” a program that has been evaluated in multiple randomised controlled trials (62–64).

As part of the intervention, participants installed two mobile companion applications: (1) the “HelloBetter MAIKI” diary and symptom tracking app, developed for this study; and (2) the “Insights” smartphone application (65). Using this technical setup, active EMA ratings as well as passive sensor and location data could be recorded during the 8-week intervention period. Therefore, we categorise our data collection into two classes: active data, which requires an interaction with the participant and passive data, which is collected automatically without a conscious interaction of the participant. The various data streams as well as the feature extraction methods are outlined in more detail below. Since MAIKI is a real-world dataset, there are also days when no data was collected of some individuals, either due to non-response or due to technical issues; section 2.3 describes the missing data handling and what criteria were applied to ensure data quality.

#### 2.1.1. Actively collected data

Active data collection took place in four different ways. First, at the beginning of the study, audio data were recorded during the SCID interview with a trained psychologist. Second, before treatment assignment, a comprehensive baseline assessment was conducted. This included sociodemographic information, as well as psychometrically validated measures of behavioral activation [Behavioral Activation for Depression Scale-Short Form; BADS-SF; (66)], anxiety symptoms [Generalized Anxiety Disorder 7; GAD-7; (67)] and quality of life [Assessment of Quality of Life; AQoL-8D; (68)]. A comprehensive list of all administered questionnaires is presented in the trial registration (cf. section 2.1). Symptom inventories were again administered at post-test (8 weeks after treatment assignment). Third, ecological momentary assessments were collected using the mobile companion application. Three times a day, participants were able to rate their affect by assessing to which extent they felt “happy” and “active” (positive affect) as well as “tense” and “sad” (negative affect). Ratings were provided on a scale from 0 (strongly disagree) to 6 (strongly agree). We derived these affect items from previous personalized sensing studies [which used the circumplex model of emotion as basis; (69, 70)] and a review of affect measurement in previous EMA studies (71), to increase the comparability with existing literature. Additionally, participants were instructed to fill out a “daily diary” at end of each day, which included a selection of items adapted from psychometric questionnaires: the Personality Dynamics Diary [PDD; agentic/communal reward, workload; (72)], CES-D scale (item 5, 7, 20), Pittsburgh Sleep Quality Index [PSQI; item 6; (73)], BADS-SF (item 1, 5, 7), as well as the PHQ-2 depression inventory [Patient Health Questionnaire, two items version; (74)]. Fourth, each patient underwent a weekly screening, in which depression [Patient Health Questionnaire, 9 item version; PHQ-9; (75)], anxiety symptoms (GAD-7), as well as perceived stress [Perceived Stress Scale, short version; PSS-4; (76)] were assessed.

#### 2.1.2. Passive mobile phone and sensor data

Using mobile phones and sensors, data was also passively collected to obtain the following information: Insights into (1) patient movement patterns through GPS data, (2) communication behaviour based on previous calls, (3) phone usage behaviour, and (4) user activity data. This exploratory feature list was selected based on technical feasibility, as well as on associations with mood symptoms found in previous studies (38). For GPS, we extracted features, such as the location variance or the daily distance travelled. Furthermore, information on spent time at home and time at specific location clusters is included. For the location clustering, the three different cluster approaches DBSCAN (77), k-means (78), and time-based clustering were used. Communication includes information about the calling patterns of study participants, e.g., how much time they spent each day making phone calls or the number of missed calls, which represent information about the frequency and entropy of calls. Phone usage contains information on typical usage behaviour in the form of frequency of phone use and total time spend on the mobile phone per day. The last group – user activity – relates, e.g., to the app usage behaviour, which provides information about the frequency of app usage. In addition, features on sleep behaviour were extracted based on the mobile phone usage data, including sleep duration and app usage behaviour during the night.

Table 1 lists all features that were extracted from the passive data of the MAIKI dataset. In total, 19 features were extracted, based on the four groups GPS, communication, phone usage, and user activity. For our experiments, we use both, all active and passive data.

**Table 1:** Features extracted from the passively collected MAIKI data.

Feature type	Name	Description
GPS	Location variance	The logarithm of the sum of the statistical variances in the latitude and the longitude of all GPS coordinates in the day
	Location entropy	The variability of the time that participants spend in significant places in the day
	Normalised location entropy	The location entropy divided by the logarithm of the number of significant places
	Time at home	Home is defined as the most frequent significant place where a participant spent the most time between 0 to 6 am; time at home is defined as the percentage of time a participant spent at home relative to other significant places
	Total distance	The total distance covered by a participant during the day
Communication	Total calling frequency	The number of times that a participant answers and makes phone calls during a day
	Total calling duration	The total time in minutes that a participant spends each day answering and making phone calls (in min)
	Non-working time calling frequency	The number of times that a participant answers and makes phone calls at times other than 8 to 6 pm during a day
	Non-working time calling duration	The total time that a participant answers and makes phone calls at times other than 8 to 6 pm during the day (in min)
	Number of missed calls	The number of calls that are marked as missed during the day
	Number of contacts	The number of contacts a participant answers and makes phone calls during the day
	Calling entropy	The variability of calling durations a participant spends in contacts during the day
	Normalised calling entropy	Calling entropy divided by the logarithm of the number of contacts during the day
Phone usage	Phone usage frequency	The number of times that a participant interacts with their phone during a day
	Phone usage duration	The total time in minutes that participants spend each day interacting with their mobile phones
User activity	Lock screen duration	The total time in minutes that participants lock their mobile phones during the day
	Number of used apps	The number of applications that a participant uses during the day
	Midnight app usage	The number of applications that a participant uses between 0 to 5 am during the day
	Sleep time	Sleep time [min] is considered to be between the last time an app was used in the previous day (or in the same day before 2 am if available) and the first time an app was used after 5 am

Four types of features have been derived: GPS, communication, phone usage as well as user activity features.

### 2.2. Data exploration

In this section, we outline the presented MAIKI dataset in more detail. A total of 65 people have participated in the study (intention to treat sample). However, a considerable percentage of the study participants only provided a small amount of data. This means that data was either only provided over a short period of time or the data was provided over a longer period of time but included many missing values. In section 2.3, we explain our pre-defined data quality criteria and how we handle missing data. Table 2 shows both statistics of the dataset: information about all participants as well as exclusively about the participants who meet our quality criteria that we use in our analyses.

**Table 2:** Statistics of the MAIKI dataset. For PHQ-2, PHQ-9 and the age of the participants, we report the (M)ean value as well as the (S)tandard (D)eviation.

Variable	All study participants		Quality-assured participants	
	M (SD) (%)	N	M (SD) (%)	N
PHQ-2	2.35 (1.61)	65	2.42 (1.62)	16
PHQ-9	10.94 (5.12)	65	11.81 (5.19)	16
Age	34.6 (11.3)	65	32.8 (8.4)	16
Female	84.6	55	81.2	13
Male	15.4	10	18.8	3

For the gender, we report the percentage of all participants. N corresponds to the absolute number of participants. All study participants are all participants that have been enrolled at the beginning of the study. Quality-assured participants are all patients that meet our pre-defined quality criteria and therefore can be used for further analyses.

**Figure 2:**
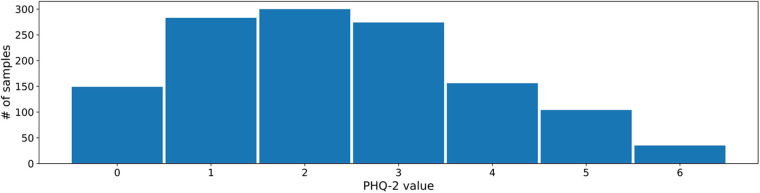
Distribution of daily self-assessed PHQ-2 ratings (scale: [0–6]) within the MAIKI study.

The 2- and 9-item Patient Health Questionnaires (PHQ) are two psychometrically valid and commonly used measures of depressive symptom severity (74, 75). Both measures are well established in clinical practice, and are therefore well suited as prediction and forecasting targets. For this reason, we choose these labels as targets for our experiments as well. Figure 2 shows the PHQ-2 label distribution in the MAIKI dataset.

**Figure 3:**
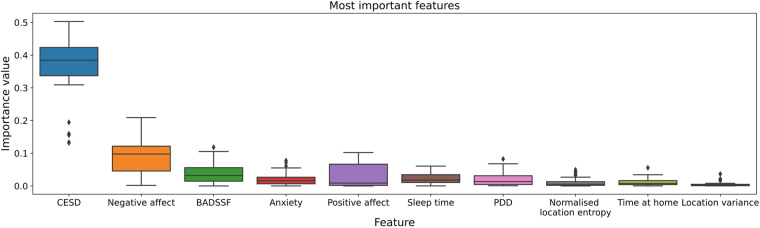
The 10 most important of all available features for PHQ-2 prediction. Determined by a Random Forest Regressor with a number of 50 different decision trees. In particular, the EMA data have a great influence on the prediction result: Center for Epidemiological Studies’ Depression Scale (CESD), negative affect, Behavioral Activation for Depression Scale-Short Form (BADSSF), Personality Dynamics Diary (PDD).

For model building, we use a broad range of active and passive data. To get an impression of which features have the greatest influence on prediction and forecasting, a random forest regressor with 50 decision trees was trained as part of the dataset exploration for predicting the PHQ-2 value. Based on this, the relevance of the different features is determined. Figure 3 shows the 10 highest variable importances of all available features identified. The analysis indicates that the EMA data in particular make a considerable contribution to the prediction result. In addition, sleep time as part of the user activity features as well as several GPS features are among the most relevant features. A detailed overview with descriptive statistics of all actively- and passively-collected data is included in the supplementary material.

### 2.3. Preprocessing

As described in section 2.2 MAIKI is a real-world dataset and therefore contains missing data. To deal with all missing values, we define and apply the following strategy and data quality criteria: (1) Each day with available features of one participant is only considered as valid if the data missing rate over all features is less than 20%. (2) A participant’s PHQ entry is only considered if the patient provides at least five days of valid data in the week prior to the corresponding PHQ label date, taking into account criterion 1 as valid data criterion. (3) Each study participant needs to provide at least 10 valid entries for PHQ-2 and 5 for PHQ-9 respectively, considering criterion 2. These criteria were imposed to maximize the number of participants who contribute information, while trying to minimize the risk of biased results due to large amounts of missing data on a person level.

If features for one day are missing, but the missing rate is less than 20%, we perform statistical imputation by calculating the mean value of the missing feature based on all available days in the week prior to the corresponding label of the participant. Criterion 3 is necessary as we need data from each participant in the training set, development set as well as test set to be able to apply our personalisation approach.

### 2.4. Baseline model

Based on our pre-defined quality criteria, we have at least five days of data for every PHQ label of every patient. To consider the sequential order of this data and to process it in the best possible way, we use a recurrent neural network (RNN) with gated recurrent units (GRUs) as a baseline model. GRUs are an improved version of a standard recurrent neural network which improve on its vanishing gradient problem using so-called update and reset gates (79). These gates are represented by vectors and can be trained to preserve long-term information without losing important parts that are relevant for prediction. The model consists of two GRU layers with a hidden size of 30, one fully connected layer with 30 neurons as well as an output layer with one neuron. Further, we use a Rectified Linear Unit (ReLU) as an activation function and apply a dropout of 20% after the fully connected layer.

We use the same baseline model for predicting depression at the end of the day as well as for one day ahead forecasting, both based on data up to 7 days before the corresponding label. This time frame was chosen so that training instances encompass each day of the week.

### 2.5. Personalisation methods

**Figure 4:**
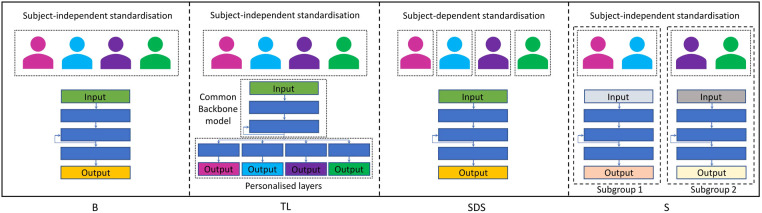
Simplified overview of our (B)aseline model as well as of the three personalisation approaches used in our experiments: (T)ransfer (L)earning with shared common layers, (S)ubject-(D)ependent (S)tandardisation, and (S)ubgroup models.

We experiment with three different personalisation methods: First, a transfer learning approach, where we have both shared common layers as well as personalised layers for each subject. Second, we use the same architecture of the baseline model, but instead of subject-independent standardisation, we apply a subject-dependent standardisation technique. As a third personalisation method, we experiment with separate subgroup models for female and male study participants. All personalisation strategies are outlined in more detail below. Figure 4 shows a simplified overview of our baseline model as well as of all three personalisation approaches.

#### 2.5.1. Transfer learning with shared common layers

Following the approach of Rudovic et al. (48) and Taylor et al. (46), we use a similar architecture by combining a common backbone model with personalised layers for each subject. In our experiments, we train at first the baseline model with data from all study participants of the train set. Afterwards, we use the two layer GRU-RNN of the pretrained model as backbone model. As personalised layers, we use the fully-connected layer with 30 neurons as well as the output layer of the baseline model, which we fine-tune separately on every individual participant, resulting in a unique model for each patient.

#### 2.5.2. Personalised subgroup models

Similar to Rudovic et al. (48), we also experiment with personalised subgroup models for depression prediction and forecasting. As criterion for subgroup models, we choose the gender information which is provided in the MAIKI dataset as shown in table 2.

For modelling of each subgroup, we use the same architecture as for the baseline model, resulting in two separate models, each trained only on data from one gender.

#### 2.5.3. Personalisation with subject-dependent standardisation

Finally, we experiment with subject-dependent standardisation. In doing so, we use the same model as for the baseline but changed the standardisation process: In all other approaches, we apply standardisation in one step for all participants in the dataset, using the statistics of the entire training population. With subject-dependent standardisation, in contrast, we apply standardisation of the data for each subject separately, resulting in one common model for all participants, but each with a personalised data standardisation.

### 2.6. Experimental setup

We design two tasks for depression prediction and forecasting: The first task is to predict the PHQ-2 score at the end of the day based on the data of the last 7 days. The second task is one day ahead forecasting of the PHQ-2 value using the data of up to the last 7 days.

For evaluating our experiments, we use 3-fold cross validation (CV). To be able to personalise a model to all participants, we need subject-dependent data splits. This means that we need, in all datasets (train/development/test), data from each patient. Therefore, we split the data from each participant in 3 folds. For the overall 3-fold CV, we use always from each participant one hold-out fold as test set, 80% data of the remaining two folds as train set, and 20% of the remaining data as development set, respectively. Furthermore, we use the mean-absolute-error (MAE) as evaluation metric as it is more robust to outliers compared to metrics such as mean-squared-error (MSE). In addition, we evaluate our results with Spearman’s ρ correlation, which measures how well models are able to predict the correct ordering of instances—thus enabling medical practitioners to detect the most severe cases.

All models were trained for 100 epochs with a batch size of 8 and a learning rate of 0.01, by gradient descent and using the Adam optimiser. The final model state was selected on the basis of validation set performance. As loss function, the MAE loss is applied.

### 2.7. Fairness metrics

A critical consideration for digital health applications is *fairness*, which requires that model predictions do not show biases for certain protected attributes like race, biological sex, or age (80). In this section, we describe the metrics we use in our experiments for analysing the fairness of personalisation methods compared to non-personalised approaches.

#### 2.7.1. Group-level fairness

With regard to fairness at group-level, we focus on biological sex as this is the only relevant, group-level variable available in our study. There is no clear definition on how to measure fairness for regression tasks, but most approaches try to achieve an equal average expected outcome for the different populations (55, 81). We adopt a similar formulation and measure fairness with two scores: the *sex fairness scores* and the *sex fairness bias* (55). These metrics are computed as follows:

(1) $$\text{Sex fairness score}={\text{MAE}}_{\text{female}}-{\text{MAE}}_{\text{male}},$$

(2) $$\text{Sex fairness bias}=\bar{{\hat{y}}_{\text{female}}-y_{\text{female}}}-\bar{{\hat{y}}_{\text{male}}-y_{\text{male}}},$$

where ${\text{MAE}}_{\text{male/female}}$ is the MAE for all male/female samples in the test set, ${\hat{y}}_{\text{male/female}}$ are the predictions for all male/female samples, $y_{\text{male/female}}$ the truth values for all male/female samples, and $\bar{\left(\cdot \right)}$ denotes the mean. The first measures the difference in MAE performance for the two sexes; a higher positive/negative score indicates a higher MAE, and thus lower performance, for males/females. The second shows whether the model systematically over- or under-predicts PHQ-2 for one of the groups (accounting for potential differences in the ground truth label distribution); a higher positive/negative bias shows that the model is systematically predicting higher PHQ-2 scores for males/females, thus showing one of the two groups as being “more depressed.” The ideal values for both metrics would be 0 – indicating a complete lack of bias in either direction.

#### 2.7.2. Individual-level fairness

A complementary fairness constraint is to ensure equal outcomes on an individual basis (55). This is important for ensuring that a depression detection system does not favour certain individuals over others. Furthermore, we expect personalisation approaches to generally improve individual-level fairness. However, there is no widely-accepted metric to quantify individual fairness. To fill this gap, we propose a set of indices motivated by related fields.

The first such index is the Gini Coefficient (GC), a typical measure of (in)equality used in the field of economics to quantify income inequality. This metric can be broadly used to quantify the diversity within a set of values; we thus co-opt for our machine-learning scenario, where we compute the GC for the individual-level performances. This essentially shows the extent to which some participants yield much higher MAE scores than others with

(3) $$\text{Gini Coefficient ( CG) }=\frac{\sum_{i=1}^{n}\sum_{\;j=1}^{n}|{\text{MAE}}_{i}-{\text{MAE}}_{\;j}|}{2n^{2}\bar{\text{MAE}}},$$

with ${\text{MAE}}_{i}$ being the performance of participant i and n the number of participants. As GC goes towards 0, the performance is mostly balanced; as it goes towards 1, a few speakers get much higher MAE scores than others.

However, GC provides only a coarse quantification of inequality. Moreover, in the present study we are primarily interested in comparing different approaches; the fact that one approach might have a lower GC than others means only that the differences amongst participants are low, but says nothing on whether the approach is overall more beneficial to those participants.

We satisfy this second criterion by computing the distance to the median participant-level MAE separately for participants with a lower/higher MAE than the median. Our rationale is as follows: The median serves as the performance that the “average” participant should expect. This divides the group of participants to a set of “winners” and “losers”; those for which performance is higher, and those for which it is lower. When comparing the sum of distances of each group to the median, we get how much each group benefits from the proposed approach. Concretely, these two indices are computed as follows:

(4) $$\text{Distance-to-Median Lower Index ( DMLI) }=\sum |{\text{MAE}}_{i}-\tilde{\text{MAE}}|,\;i\in [1,n]\;:\;{\text{MAE}}_{i}<\tilde{\text{MAE}}$$

and

(5) $$\text{Distance-to-Median Upper Index ( DMUI) }=\sum |{\text{MAE}}_{i}-\tilde{\text{MAE}}|,\;i\in [1,n]\;:\;{\text{MAE}}_{i}>\tilde{\text{MAE}},$$

where MAE and n are again the performance per participant and the number of participants, respectively.

The use of DMLI and DMUI allows for a more nuanced selection between different methods. For example, practitioners might select to optimise for DMUI (the lower the better)—thus capping the worst-case scenario. Others might choose to optimise for DMLI (the higher the better)—thus boosting performance for those participants for which the system works satisfactorily. This choice (which is reminiscent of the precision-recall tradeoff) is context-dependent.

## 3. Results

### 3.1. Prediction of daily PHQ-2 scores

Table 3 shows the results of the performed experiments. In the case of PHQ-2 prediction, the personalised models clearly outperform the non-personalised baseline. The best result is obtained using subject-dependent standardisation which yields a MAE of .801 and Spearman’s ρ correlation of 0.728 compared to the non personalised model that achieves 1.062 and .604, respectively. The other two personalisation methods also improved the result, although not quite as strongly as the subject-dependent standardisation method. In addition to the global Spearman’s ρ correlation coefficient, the local Spearman’s ρ also improved from 0.431 using the baseline to an average of 0.473 across the different personalisation strategies. In depression prediction, e.g., predicting PHQ-2 with heterogeneous health data, personalisation can therefore add value and improve performance compared to non-personalised approaches.

**Table 3:** PHQ-2 (scale: [0–6]) prediction and forecasting results, reported as mean-average-error (MAE) and Spearman’s ρ correlation using the four methods: (B)aseline-GRU without personalisation, (T)ransfer (L)earning, (S)ubgroup models, and (S)ubject-(D)ependent (S)tandardisation.

Method	Prediction		Forecasting	
	MAE	ρ	MAE	ρ
B	1.062	0.604	1.539	0.105
TL	0.990	0.668	**1.349**	**0.349**
S	0.978	0.666	1.465	0.271
SDS	**0.801**	**0.728**	1.496	0.254

### 3.2. One day ahead-forecasting of PHQ-2 scores

In one day ahead forecasting of PHQ-2 scores, the different personalisation methods improve the baseline result as well. Table 3 shows the results for PHQ-2 forecasting. The best MAE and Spearman’s ρ is obtained using the personalised transfer learning approach with a shared common backbone model and personalised layers with a result of 1.349 and 0.349 compared to the baseline which yields 1.539 and 0.105, respectively.

### 3.3. Group-level fairness

Table 4 presents the *sex fairness score* as well as the *sex fairness bias* for each method. The baseline shows a high bias for both tasks; the performance for females is consistently higher than for males (i.e., the Sex Fairness Score is negative) while females are being systematically predicted as having higher PHQ-2 scores (i.e., the Sex Fairness Bias is negative). Collectively, these two metrics show that males are predicted more wrongly with a bias towards negatives—causing a lot of high PHQ-2 cases to be mispredicted as having low PHQ-2 scores. Notably, personalisation methods most often improve on both metrics, with subgroup-models showing the best performance for prediction (where the bias is almost completely eliminated), and transfer learning with shared layers performing best for forecasting (where the performance still remains higher for females but there are no systematic over-/under-predictions).

**Table 4:** Sex-Fairness-(S)core and Sex-Fairness-(B)ias of the different methods for PHQ-2 prediction and forecasting: (B)aseline-GRU without personalisation, (T)ransfer (L)earning, (S)ubgroup models, and (S)ubject-(D)ependent (S)tandardisation.

Method	Prediction		Forecasting	
	S	B	S	B
B	−0.460	0.576	−0.369	0.630
TL	−0.291	0.066	**−0.279**	**0.051**
S	**0.055**	**−0.065**	−0.449	−0.081
SDS	−0.112	0.127	−0.330	0.275

### 3.4. Individual-level fairness

**Figure 5:**
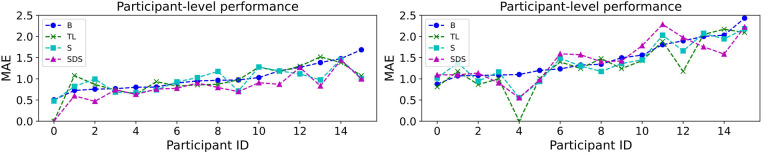
Individual-based MAE performance for PHQ-2 prediction (left) and forecasting (right) using the (B)aseline model as well as the three personalisation methods: (T)ransfer (L)earning, (S)ubgroup models, and (S)ubject-(D)ependent (S)tandardisation. The MAE values are calculated using the samples of each participant of the study. The participants are sorted from best to worst performance of the baseline model.

Similar to Wagner et al. (55), we present the individual-level fairness by considering the MAE performance separately for each subject in the first step. This is done by computing MAE using the samples of each participant. The outcome is visualised in figure 5. Performance shows big fluctuations across different participants. For example, for the prediction task with transfer learning, it ranges between [0.000, 1.519]—a large difference which means that the system works much better for some individuals over others. Interestingly, there is sometimes disagreement as to where the system works well (e.g., for forecasting, participant #4 is ranked as the best performing one for all methods except the baseline). This is an aspect of the underspecification exhibited by machine learning architectures, where different models trained on the same data show different behaviours on distinct subpopulations of it (55, 82).

Table 5 shows all three indices proposed in section 2.7.2: GC, DMLI, and DMUI. We note that the baseline model is already showing good behaviour with respect to GC; in fact, it comes first for forecasting with a GC of 0.161 and second for prediction with a GC of 0.166 (following subgroup models with 0.148). This shows that our proposed personalisation approaches increase the diversity of performance within individual participants, thus seemingly increasing inequality. However, the other two indices show a different pattern. DMLI is consistently higher when personalisation is used, while DMUI is consistently lower—this shows that “winners” further diverge from the median while “losers” come closer to it. Overall, this leads to a trend where all participants gain by having improved MAE scores. The fact that GC decreases is side-effect of the fact that DMLI increases more than DMUI decreases (on average). This means that all three personalisation methods favour “winners” (participants who fare better than the median). This leads to a bigger divergence between those on the upper and those on the lower end.

**Table 5:** Individual-level fairness captured by the Gini Coefficient (GC), Distance-to-Median Lower Index (DMLI), and Distance-to-Median Upper Index (DMUI). GC serves as a coarse marker of inequality, with DMLI and DMUI further elucidating whether the benefits are reaped by those in the lower or upper end of individual-level performance.

Method	Prediction			Forecasting		
	GC	DMLI	DMUI	GC	DMLI	DMUI
B	0.166	1.451	2.311	0.161	1.704	3.890
TL	0.188	1.910	2.136	0.224	2.664	3.798
S	0.148	1.859	1.374	0.179	2.497	3.248
SDS	0.203	1.611	1.730	0.182	3.377	2.800

As the choice of optimising for DMUI or DMLI is context-dependent, we avoid marking one of the approaches as “best”: transfer learning shows better behaviour for DMLI, whereas the other two work better for DMUI.

## 4. Discussion

In this study, we investigated the performance benefits of different personalisation strategies (transfer leaning, subgroup models, subject-dependent standardisation) in predicting individuals’ (future) depressive symptom severity as measured by the PHQ-2. Our experiments were based on a novel dataset that was collected while patients with elevated depressive symptoms received a digital intervention under routine care-like conditions. In a subset of patients who provided sufficient active EMA as well as passive mobile sensor data, we found that all investigated personalisation strategies lead to improved predictions of the end-of-day depressive symptom severity (MAE=0.801 to 0.990), compared to a general RNN model (MAE=1.062). A similar pattern was found for forecasts of patients’ depressive symptoms the next day (MAE=1.349 to 1.496; baseline model: MAE=1.539). We also examined the models’ fairness with respect to patients’ self-reported biological sex, which is a crucial desideratum in digital mental health applications. We found that personalisation was able to reduce the bias inherent in the initial baseline model (in our case favoring females). Similarly, we found that all patients obtain improved predictions via personalisation, although not all benefit equally. This result is in line with previous findings by Jacobson and colleagues (39), who found that the perfomance of idiographically weighted models in predicting depressed mood varied substantially between individuals. Our findings also corroborate the results of Taylor et al. (46), who report that personalized Multitask Learning (shared common layers neural network, multi-kernel learning using support vector machines, and hierarchical Bayesian models with a common Dirichlet process prior) improved prediction accuracies by 11–21% over non-personalized models. However, in contrast to aforementioned study, we only found that two personalisation strategies (Subject-Dependent Standardisation and Transfer Learning) resulted in substantial benefits compared to the non-personalised baseline RNN.

Revisiting the hypothesis of Taylor and colleagues (46), one potential reason why model personalisation may provide benefits over “traditional” machine learning methods is because this allows to deal with *heterogeneity*, a feature that is characteristic both of depression and the way patients respond to treatment (83, 84). Personalisation allows models to learn patterns that may be specific to the symptomatology of each patient, which could explain why performance benefits arise. From a clinical perspective, the ability to generate tailored forecasts of the depressive symptom severity is very helpful. This could allow to react pre-emptively to short-term symptom changes during treatment [e.g., sudden gains or depression spikes; (85, 86)], for example by providing personalised feedback, therapeutic recommendations, or direct contact to health care professionals.

Several limitations and challenges should be considered. First, due to missing values, only a subset of patients in the MAIKI dataset could be included in our experiments. This is a common finding in studies based on real-world data, particularly if mobile sensor features are included [see, e.g., (34, 35)]. Seamless recording of sensor data proved to be technically challenging in many cases, given that all patients used their privately owned smartphone device. Some individuals, for example, reported difficulties installing and navigating the companion tracking app. Seamless tracking of passive features could not be ensured on some devices due to software issues. These problems might be mitigated in future studies by providing participants with mobile devices or wearables; however, this could compromise the ability to implement developed models into routine care, where provision of standardised devices is typically not possible. Second, in our exploration of variable importances, we found that most of the important features were based on actively assessed data. In a related study, it could be shown that reasonable depressive symptom severity forecasts could also be generated using actively obtained EMA data only (47). Therefore, focusing on actively assessed data may be a way to provide personalised depression forecasts at greater scale, given the lower technical requirements. Structured self-monitoring is a common feature in cognitive-behavioural treatments for depression (87), and EMA ratings may therefore serve a twofold objective: providing therapeutic feedback to patients, while at the same time allowing to build a personalised forecasting model of the individual’s symptomatology.

Another limitation is that some of the personalised models investigated in this study require that several weeks of individual training data are available. This represents a constraint in clinical practice, since forecasts may only become available a few weeks into treatment. Future studies may examine how much the “burn-in” phase for individual patients can be shortened while still retaining appreciable forecasting performances. Alternatively, general models may be used initially, switching to a personalised model once sufficient patient data has been collected. It is also important to note that a wide variance of PHQ values must be collected from each participant during this time—otherwise, the personalised model may tend to overfit. To further counteract this, we also propose methods that incorporate features from the whole population, such as transfer learning with shared common layers, as well as models that are personalised on smaller subgroups rather than just a single individual.

In sum, our findings provide preliminary evidence that model personalisation, based on EMA ratings and supported by passive mobile data, can be used to improve daily forecasts of depressive symptom severity in real-world patient populations, as well as their fairness. Our experiment follows a broader research trend, found both in affective computing and clinical psychology, in which greater emphasis is put on individual patterns and dynamics of psychopathology [see, e.g., (41, 46, 88)]. Further research is needed to corroborate our results in larger samples, and to develop model personalisation strategies that can be implemented into digital mental health care at scale.

## Data Availability

Factually anonymized scientific use files containing data obtained as part the MAIKI study are available to researchers of scientific institutions. Data may only be analysed for non-commercial purposes based on a data use agreement. Due to confidentiality reasons, the scientific use files contain fewer and further aggregated variables. Requests to access the datasets should be directed to e.heber@hellobetter.de.
