# Supplementary material for: Personalised depression forecasting using mobile sensor data and ecological momentary assessment
Source: Front Digit Health. 2022 Nov 18;4:964582. doi: 10.3389/fdgth.2022.964582 (PMC9715619; doi:10.3389/fdgth.2022.964582)
Supplement: Supplementary file 1 [file Datasheet1.pdf]

Supplementary Material

1 DATASET STATISTICS

Table S1. Descriptive statistics of actively-collected data as well as passively-collected (GPS, Communication, Phone usage, User activity) data. For each feature, we report the (M)ean value, (S)tandard (D)eviation and the (F)requency at which the data was collected.

Data type	Name	M	SD	F
Actively collected data	Anxiety	2.20	1.99	daily
	BADS-SF (item 1, 5, 7)	10.71	4.04	daily
	CES-D (item 5, 7, 20)	7.49	4.04	daily
	GAD-7	9.64	4.59	weekly
	Negative affect	2.38	1.46	daily
	PDD (item 1, 3, 4)	10.47	3.66	daily
	Positive affect	2.88	1.29	daily
	PSQI (item 6)	2.75	1.55	daily
	PSS-4	8.45	2.86	weekly
GPS	location variance	-9.32	4.80	daily
	location entropy	0.44	0.37	daily
	normalised location entropy	0.33	0.28	daily
	time at home	0.73	0.29	daily
	total distance	10.33	11.94	daily
Communication	total calling frequency	2.77	3.06	daily
	total calling duration	16.12	27.45	daily
	non-working time calling frequency	0.69	1.43	daily
	non-working time calling duration	5.02	16.44	daily
	number of missed calls	0.47	0.86	daily
	number of contacts	1.71	1.34	daily
	calling entropy	0.20	0.34	daily
	normalised calling entropy	0.20	0.33	daily
Phone usage	phone usage frequency	58.80	46.20	daily
	phone usage duration	252.16	185.79	daily
User activity	lock screen duration	13.56	36.93	daily
	number of used apps	156.60	141.76	daily
	midnight app usage	3.24	4.05	daily
	sleep time	470.13	86.55	daily
